# Insights into neurosteroids and their role in women with epilepsy

**DOI:** 10.3389/fgwh.2024.1363470

**Published:** 2024-06-10

**Authors:** Lata Vadlamudi, Daniel Paul Ashley, P. Emanuela Voinescu

**Affiliations:** ^1^ UQ Centre for Clinical Research, The University of Queensland, Brisbane, QLD, Australia; ^2^ Department of Neurology, Royal Brisbane & Women’s Hospital, Brisbane, QLD, Australia; ^3^ The Ochsner Clinical School, Ochsner Health, New Orleans, LA, United States; ^4^ Faculty of Medicine, The University of Queensland, Brisbane, QLD, Australia; ^5^ Department of Neurology—Division of Epilepsy, Division of Women’s Health, Brigham and Women’s Hospital, Harvard Medical School, Boston, MA, United States

**Keywords:** women, neurosteroids, epilepsy, seizure, progesterone, sex steroids, oestrogen, GABA-A receptor

## Abstract

Epilepsy, is a serious neurological condition, characterized by recurring, unprovoked seizures and affects over 50 million people worldwide. Epilepsy has an equal prevalence in males and females, and occurs throughout the life span. Women with epilepsy (WWE) present with unique challenges due to the cyclical fluctuation of sex steroid hormone concentrations during their life course. These shifts in sex steroid hormones and their metabolites are intricately intertwined with seizure susceptibility and affect epilepsy during the life course of women in a complex manner. Here we present a review encompassing neurosteroids—steroids that act on the brain regardless of their site of synthesis in the body; the role of neurosteroids in women with epilepsy through their life-course; exogenous neurosteroid trials; and future research directions. The focus of this review is on progesterone and its derived neurosteroids, given the extensive basic research that supports their role in modulating neuronal excitability.

## 1 Neurosteroids

### 1.1 Neurosteroid synthesis

#### 1.1.1 Distant synthesis

Traditionally sex steroids are produced by endocrine regulation and feedback by the hypothalamic-pituitary-ovarian axis. The hypothalamus is involved with regulation, production, and pulsatile secretion of gonadotropin-releasing hormone (GnRH), which then controls the release of follicle-stimulating hormone (FSH) and luteinizing hormone (LH) from the pituitary gland. These hormones induce ovulation, stimulating estradiol and progesterone production that provides feedback to neuronal cells, in particular the temporo-limbic system (amygdala, hippocampus) (1). This is a well-established pathway whereby steroids synthesized in the distant endocrine glands, cross the blood brain barrier, and then act directly in the brain.

#### 1.1.2 Local synthesis

There is a second mechanism which is *de novo synthesis* in the brain. French endocrinologist Etienne-Emile Baulieu first introduced the term neurosteroids in 1981 to describe steroids produced in the brain ‘*de novo*” (2), based on the identification of steroids accumulating in rat brain, independent of the traditional endocrine pathway. Animal models demonstrated neurosteroid concentrations higher in the nervous system compared with plasma concentrations. It has been well established with animal models that the neurosteroid biosynthetic enzymes are also present within the human brain and that precursor steroids are produced *de novo* in glial cells and neurons (3). There is complexity of neurosteroid biosynthetic enzymes, with regards to region and cell type-specific expression as well as developmental regulation of these enzymes (4).

There are 3 distinct steps in *de novo* synthesis of neurosteroids (NS). The first is that cholesterol is directed to the outer mitochondrial membrane by the steroidogenic acute regulatory (StAR) protein; StAR is complexed with the translocator protein (TSPO) on the outer membrane of the mitochondria, which then mediates cholesterol transport from the outer mitochondrial membrane to the inner membrane; and finally the cytochrome P450 side-chain cleavage (P450scc) enzyme on the inner mitochondrial membrane creates the precursor pregnenolone (5).

The term “neuro-active steroids” has been utilised for locally synthesized steroids, which can be brain derived or from systemic precursors (6). For the scope of this review, we will refer to all sex steroids and their derivatives, produced both distally and locally in the brain, as “neurosteroids” (NS), as their combined actions are of interest from a clinical perspective.

**Figure 1:**
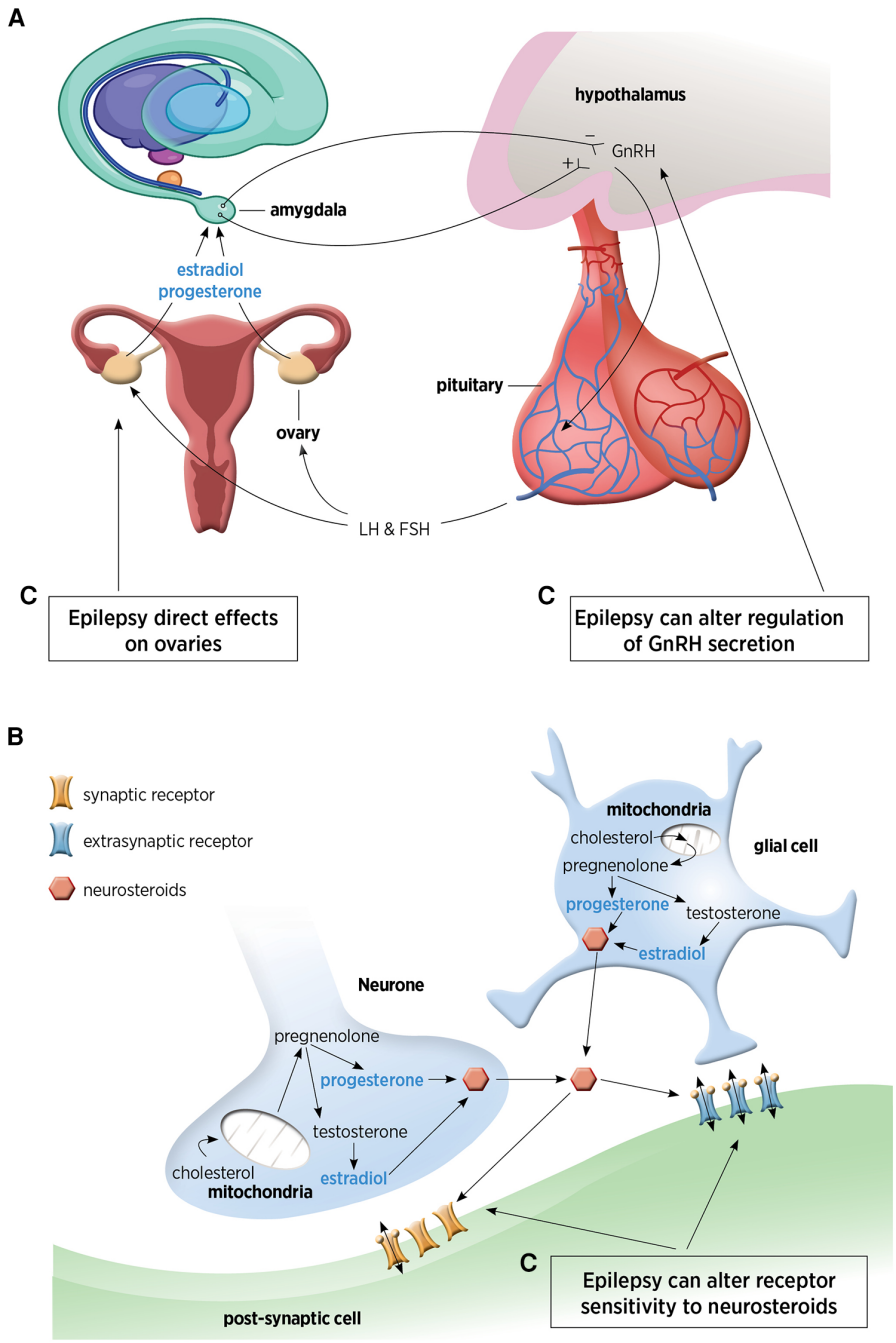
(**A**) Hypothalamic-pituitary-ovarian axis responsible for distal steroid hormone production. This figure has been adapted from Tauboll et al. (1). (**B**) Neuronal and glial pathways for local neurosteroid production within the brain. (**C**) The complex inter-relationship between epilepsy and neurosteroids. GnRH, gonadotrophin releasing hormone; LH, luteinizing hormone; FSH, follicle-stimulating hormone.

Figures 1A,B demonstrates the sites of production of NS.

### 1.2 Neurosteroid regulation

Synthesis of NS occurs in neurones and glia and is controlled by the translocator protein (TSPO) (3). From vertebrate studies, it is thought that neuropeptide control, such as gonadotropin-releasing hormone (GnRH) may also regulate NS biosynthesis as GnRH is expressed in the brain, outside of the hypothalamus and pituitary (5). In females, pulsatile GnRH secretion regulates estradiol synthesis in ovaries and NS in the hippocampus, highlighting the basis for the cyclical nature of NS production in women (7) and why NS production fluctuates with the ovarian cycle (8). The GnRH-induced rise in estradiol establishes a connection between the hypothalamus and the hippocampus, potentially underpinning the cyclical modulation of spine density in the female hippocampus (9).

**Figure 2:**
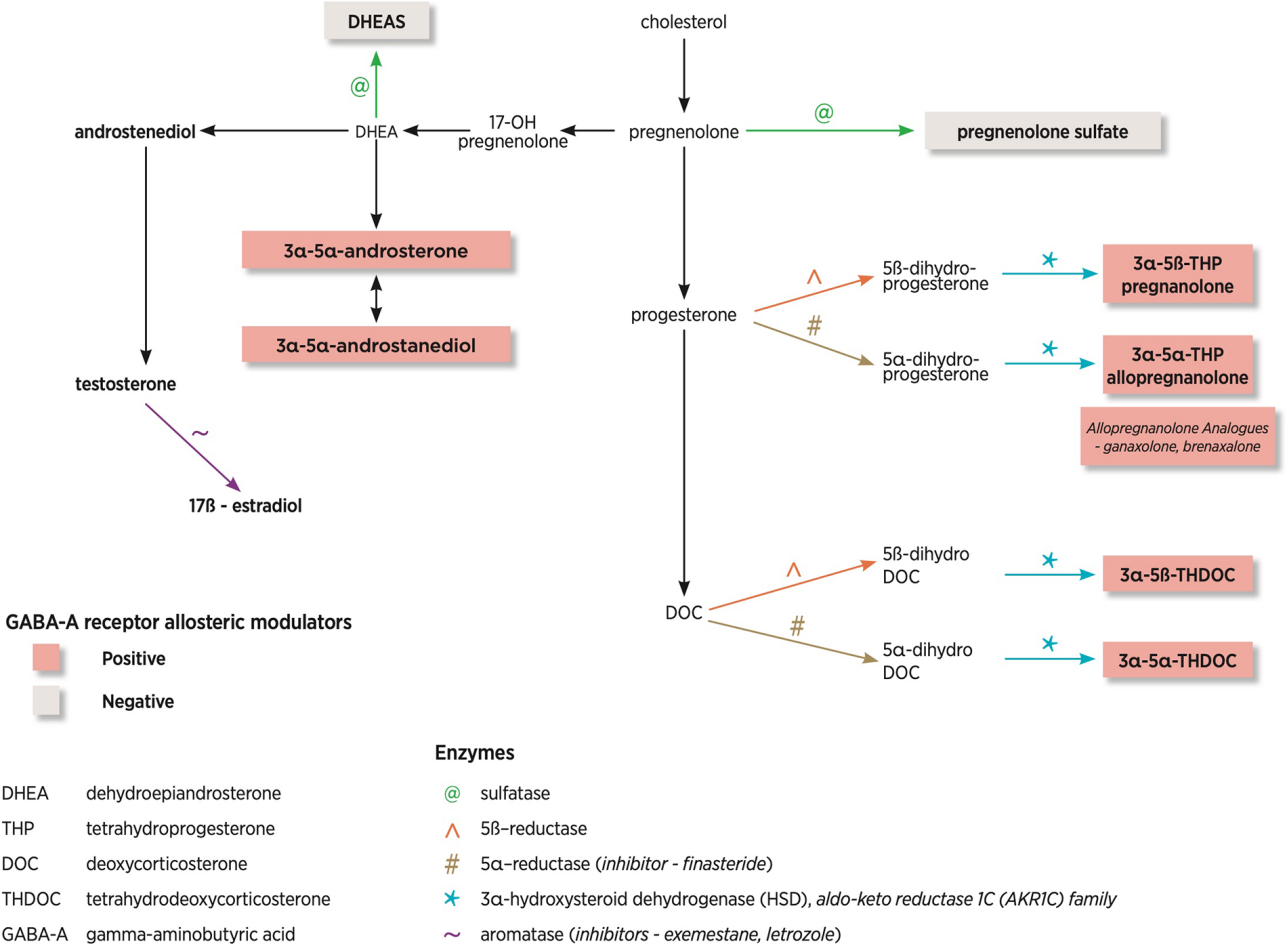
Neurosteroid pathway and enzymes involved. This diagram has been adapted from Finn and Jimenez (10).

Figure 2 demonstrates the NS pathway and the most common enzymes involved as well as differing modulation on the GABA-A receptor. The neurosteroids derived from deoxycorticosterone are tetrahydrodeoxycorticosterones (THDOC), which are part of the hypothalamic-pituitary-adrenal axis. This represents a more distant pathway compared with the direct progesterone-derived neurosteroids. Also of note is the reduction of testosterone by aromatase leads to the generation of 17-β estradiol.

### 1.3 Neurosteroid mechanisms of action

The mechanism of action differs for each NS. One mechanism being non-genomic mechanisms where the sex steroids act on membrane ion channels/receptors (rapid action, within minutes) and the other mechanism being genomic, where the sex steroid hormones act in the nucleus and alter mRNA transcription (delayed action) (6).

#### 1.3.1 Progesterone

The largest body of literature is on progesterone-derived NS and their action on the GABA-A receptor (membrane action). The GABA A-type receptor represents a pentameric protein` with five protein units, including 2α and 2β sub-units and one subunit of either δ, γ and ε, θ, π. Some of the protein subunits have multiple isoforms (α1–α6, β1–β3 and γ1–γ3) (11). The α1, β2 γ2 is the most common subunit within the brain and different sub-unit compositions are observed in different brain regions (11).

The opening of the GABA ion channel allows for movement of chloride ions across the cell membrane following a gradient and this may lead to hyperpolarization, pending movement of the chloride ions. This receptor modulates most of the inhibitory neurotransmission in the brain through synaptic (phasic) and extrasynaptic (tonic) inhibition (3). The extra-synaptic GABA-A receptors are the key target for NS (12).

NS are the most potent modulators of δ-GABA-A receptors (13). If a specific NS binds to the δ-GABA-A receptors, this can lead to an increased affinity for GABA, termed a positive allosteric modulator. Higher NS concentrations can also lead to activation of GABA-A receptors in the absence of GABA. There are positive, allosteric modulators of the GABA-A receptor (allopregnanolone, THDOC, and androstanediol), which enhance the GABAergic response and negative, allosteric modulators (DHEA, pregnenolone) which suppress the GABAergic response. Figure 2 shows that NS can be positive or negative modulators of the GABA-A receptor pending on the steroid molecule structure. Positive modulators of GABA-A receptors are anti-convulsant whilst negative modulators are pro-convulsant.

Progesterone also activates the progesterone receptors (genomic mechanism), isoforms A and B, expressed in the brain, and especially in the hippocampal neurons, and their expression is regulated by estrogen (14). Joshi and colleagues demonstrated that progesterone receptor activation also increased expression of GluA1 and GluA2 subunits of AMPA receptors in the hippocampi (15). These ﬁndings shifted the dominant paradigm of progesterone as a solely anticonvulsant agent and reveals that it has dual actions on the brain: inhibition via NS and excitation via the progesterone receptor.

#### 1.3.2 Estrogen

There also exists rapid, membrane-initiated, 17*β*-estradiol mediated actions in addition to the classic nuclear signaling pathway. 17β-estradiol acts as a posttranscriptional modulator of excitatory NMDA receptors (16). Estrogen activates estrogen receptors, isoforms α and β, and enhances the expression of the GluA1 subunit of AMPA receptors via activation of estrogen receptor β (17). Compared to progesterone metabolites that act predominantly on GABA-A receptors, membrane-mediated effects of 17β-estradiol trigger various intracellular cascade pathways, leading to changes in ion channel regulation and neuronal excitability (18).

17β-estradiol also facilitates neuroprotection, synaptic and cognitive preservation, has anti-inflammatory effects and regulates microglial activation and function (19). The is evidence for interactions between 17β-estradiol and the signalling molecule brain-derived neurotrophic factor (BDNF) (20). Experimental models have also demonstrated increased concentrations of BDNF in the hippocampus, has both a protective effect as well as increased excitability (21).

A potential mechanism to explain the effect of estradiol on hippocampal excitability was demonstrated in a rat model, where spine density in excitatory postsynaptic synapses positively correlated with estradiol concentrations (22). This work could have implications for cognitive performance not only during the menstrual cycle but also during the menopausal transition and later in life.

### 1.4 Neurosteroids and epilepsy

#### 1.4.1 Altered expression of GABA-A receptors due to seizures

Multiple studies have similarly concluded that altered GABA-receptor expression in epileptic animals is the basis for the reduced NS sensitivity, evidenced by reduction in δ and α1 subunits and increased α4 and γ1 sub-units (23). This has been demonstrated by NS at physiological concentrations failing to lead to increased synaptic and tonic inhibition in epileptic animals. Joshi demonstrated reduced δ- subunit-containing GABA-A expression and upregulation of γ2 subunit following status epilepticus (23).

#### 1.4.2 Altered expression of GABA-A receptors due to neurosteroids

In late diestrus cycle of the mouse (high-progesterone phase), there was increased δ subunit-containing GABA-A receptors and decreased γ2 subunit-containing GABA-A receptors (24). This enhanced expression of δ subunit-containing GABA-A receptors increased tonic inhibition, reducing neuronal excitability and decreased seizure susceptibility (24). This study also found that by eliminating the cycling of δ GABA-A receptors by antisense RNA treatment or gene knockout, changes in excitability were prevented and hypothesized that cyclical seizures (as seen in catamenial epilepsy) maybe due to abnormalities in regulation of the normal cycling of δ subunit-containing GABA-A receptors (24).

#### 1.4.3 The complex interplay of neurosteroids and seizures

There is a complex inter-relationship between NS and epilepsy, with epilepsy affecting multiple sites in the NS biosynthesis process (as shown in Figure 1C). Epilepsy has direct effects on the ovaries, can alter GnRH regulation (1, 9) and can also alter receptor sensitivity to NS (as described earlier).

#### 1.4.4 Animal studies

Over the past few decades, there has been extensive research into NS using preclinical models to explore their implications in depression, anxiety, and excitability disorders. Miziak and colleagues provide a comprehensive table summary of animal models demonstrating the anticonvulsant and proconvulsant actions of the different NS, based on their particular chemical structure (25).

## 2 The role of neurosteroids in women with epilepsy across their life-course

### 2.1 Pre-puberty

In Protocadherin 19 in female epilepsy (PCDH19-FE), seizure onset and offset coincides with periods of changes in NS (26). Seizure onset occurs after mini-puberty (around 8 months of age), which seems to start after the fall of *in utero* NS concentrations. The median age of onset was 8 months old, while the median age of offset was 12 years old, when NS are elevated in association with puberty (26).

This landmark publication reviewed transcriptomics in PCDH 19-FE patients and identified 94 dysregulated genes, involved with NS metabolism. Interestingly, nearly half of the genes demonstrated gender-biased expression when compared with transmitting males (26).

Of particular interest within the dysregulated gene set were the genes *AKR1C2* and *AKR1C3*, members of the aldo-keto reductase 1C (AKR1C) family (AKR1C1-4), of which only *AKR1C1-3* is expressed in the brain (26). These enzymes are responsible for reducing NS into downstream metabolites such as allopregnanolone. AKR1C3 gene encodes steroid hormone-metabolizing enzyme, 3α-HSD. Figure 2 demonstrates the location of this enzyme in the biosynthetic pathway. They found that *AKR1C3* mRNA and 3α-HSD protein levels were significantly reduced in PCDH19-FE (26). To further support their hypothesis, they reviewed blood allopregnanolone concentrations, which were also reduced, highlighting the potential role of NS in PCDH19-FE (26).

### 2.2 Childbearing Age

There is growing evidence for the key role of NS in catamenial seizure exacerbation. It is the cyclical changing balance of sex hormone concentrations that drives an increased seizure frequency with certain menstrual/ovarian cycle phases. There are three common patterns C1 (around menstruation); C2 (ovulation); C3 (anovulatory cycles from mid-cycle to menstruation). Building upon clinical observations, the concept of treatment with cyclical progesterone was conceived leading to the only NIH-approved clinical trial to-date to test a hormonal treatment for catamenial epilepsy (27). This study, led by Herzog, yielded inconclusive results for women with focal catamenial epilepsy overall. However, sub-group analysis demonstrated superior efficacy in women with a prominent C1 pattern (3-fold increase in seizure frequency around menstruation) (27, 28). Herzog's subsequent investigation implicated allopregnanolone as the mediator of seizure reduction in progesterone-treated women (29). Notably, the trial employed progesterone and not allopregnanolone. The previously describer proconvulsant effect via the progesterone-derived NS nuclear action may play a role in seizure exacerbation (15). A more recent pilot study of 173 menstrual cycles from 23 women with epilepsy demonstrated that 52.2% of women met the criteria for one or more catamenial pattern (30), further supporting the role of NS in seizure exacerbation.

### 2.3 Pregnancy

A novel study during pregnancy study demonstrated that lower allopregnanolone concentrations were associated with increased seizure frequency (31).

### 2.4 Peri-menopausal and menopause

Harden reviewed the effect of menopause and perimenopause on the course of epilepsy and in women with a history of catamenial epilepsy, seizures increased during perimenopause in and decreased at menopause (32). This data re-affirming the role of fluctuating hormones on seizure control during perimenopause and then reduction in seizures when hormones no longer fluctuate during menopause.

## 3 Exogeneous neurosteroid trials

### 3.1 Aromatase inhibitors- exemestane and letrozole

The enzyme aromatase and where it acts in the biosynthetic pathway is shown in Figure 2. A clinical case report demonstrated seizure reduction with tamoxifen and complete seizure freedom with the aromatase inhibitor exemestane (given for the management of breast cancer in a post-menopausal woman), highlighted the link between hormones and seizure control (33).

Harden and MacLusky reported a case report of a 61-year-old man with temporal lobe epilepsy who was commenced on letrozole (aromatase inhibitor) off-label to improve libido and energy levels. There was sustained improvement in seizure control, seizure exacerbation with withdrawal of letrozole and subsequent improvement with re-commencement, once again highlighting the link between hormones and seizure control (34).

### 3.2 Reductase inhibitor- finasteride

Finasteride is a 5α reductase enzyme blocker, which blocks the conversions of progesterone and deoxycorticosterone to pregnanolone, allopregnanolone and THDOC. Figure 2 highlights where in the biosynthetic pathway this enzyme plays a role. A case report of a woman by Herzog and Frye demonstrated that finasteride (commenced for male pattern baldness) increased seizures, which had been controlled with progesterone (35).

### 3.3 Ganaxolone (3β-methylated analog of allopregnanolone)

Natural NS such as allopregnanolone have low bioavailability due to rapid inactivation, however synthetic NS, such as ganaxolone may overcome these limitations (3). Ganaxolone (ZTALMY®; Marinus Pharmaceuticals) is a positive allosteric modulator of the GABA-A receptor (36). Phase 1 trials were undertaken in 1994 and orphan drug status was achieved for PCDH19-FE, status epilepticus and fragile X syndrome (36).

A phase II trial of two patients were given oral ganaxolone from day 21 of their menstrual period until 3 days after their menstrual period for 4 months demonstrated a decrease in total seizure burden with cessation of perimenstrual seizure activity. This promising outcome is yet to be replicated with a larger sample size and longer follow-up (37).

Safety and efficacy of galaxolone was demonstrated in a double-blind phase of the randomized, placebo-controlled phase III trial in patients with cyclin-dependent kinase-like 5 (CDKL5) deficiency disorder, leading to the first FDA approval of ganaxolone (38).

### 3.4 Brenaxolone (aqueous formulation of allopregnanolone)

A phase III trial of brenaxolone was undertaken for refractory status epilepticus. The primary endpoint did not differ significantly from placebo at the end of the double-blind period (39). During the open label extension, 37% of patients on brexanolone achieved treatment response (39).

### 3.5 PF-06372865 (selective GABA-A receptor positive allosteric modulator)

This is a positive allosteric modulator of α2/3/5 subunit-containing GABA-A receptor. A phase 2A double-blind study of seven patients with photoparoxsymal response demonstrated a statistically significant suppression of the photosensitivity response compared with placebo, with similar responses to lorazepam, highlighting the potential of a selective GABA-A positive allosteric modulator (40).

### 3.6 Hormone replacement therapy

A randomized, double-blind, placebo-controlled study limited in numbers (*n* = 21), due to the outcomes from the Women's Health Initiative, reviewed women taking Prempro (0.625 mg of conjugated equine estrogens plus 2.5 mg of medroxyprogesterone acetate) daily, or double-doses for a 3-month treatment period (41). The outcomes demonstrated a dose-related increase in seizure frequency in post-menopausal women (41).

## 4 Further research directions

There is solid evidence that NS modulate neuronal excitability. Yet, there is much to be understood about their complex actions in the brain and the inter-relationship between NS and epilepsy. While there are many research avenues to pursue, this relationship is easier to measure in WWE during their life course. There are the cyclic monthly fluctuations during reproductive years; the extreme effects of high NS concentrations during pregnancy; and low NS concentrations at menopause.

There is an enormous knowledge gap at the clinical level. We hypothesize the potential role of exogenous NS administration to improve cyclical seizure exacerbation in patients with catamenial epilepsy; for the pregnant women to reduce seizure and medication burden to the mother and foetus; and for peri- and post-menopausal women to reduce seizure burden and improve cognition.

At the basic science level, we hypothesize that seizure susceptibility can be influenced by: (1) NS concentrations, influenced by multiple variables: synthesis capacity, genetics, biological stage; (2) GABA-A receptor expression and sensitivity, with a different degree of influence based on the location of the epileptogenic focus.

From a clinical science perspective, the only placebo-control blinded study employed a pulse progesterone treatment in a population of women with catamenial seizure exacerbation. It failed to show an impressive benefit, except for patients with significant perimenstrual worsening, yet the timing of the treatment in this trial would not be expected to clearly be helpful for other catamenial patterns. Moreover, the lack of an impressive response is not surprising now that we learned about the progesterone dual actions on excitability. However, there are already synthetic allopregnanolone analogues available and some are FDA-approved for other indications. The trials to assess for their efficacy in epilepsy were not tailored to a patient population where one would expect a benefit (for example catamenial epilepsy), but rather to status epilepticus patients where seizures are provoked by different factors or to very heterogenous groups of refractory epilepsy patients. In the absence of a dedicated treatment option, many clinicians employ continuous progestins or combined contraceptive pills to suppress sex hormone fluctuation, a strategy that they know works for many, despite the lack of solid clinical evidence for it.

At the other end of the life-course, peri-menopause and menopause remain a largely unmapped territory. The essence is to better understand the complex relationship between changing NS concentrations and seizure burden. This will enable greater insights into the pros and cons of exogenous hormone replacement in women with epilepsy.

The role of NS in epilepsy, particularly in the context of women’s health, represents a fascinating and uncharted field of research. The potential for advancements in understanding NS molecular underpinnings holds great promise for developing more personalized treatments, to enhance clinical outcomes for women with epilepsy.
